# Circulating T_FH_ Subset Distribution Is Strongly Affected in Lupus Patients with an Active Disease

**DOI:** 10.1371/journal.pone.0075319

**Published:** 2013-09-19

**Authors:** Carole Le Coz, Aurélie Joublin, Jean-Louis Pasquali, Anne-Sophie Korganow, Hélène Dumortier, Fanny Monneaux

**Affiliations:** CNRS, Institut de Biologie Moléculaire et Cellulaire, Immunopathologie et Chimie Thérapeutique/Laboratory of excellence MEDALIS, Strasbourg, France; Institut National de la Santé et de la Recherche Médicale U 872, France

## Abstract

Follicular helper T cells (T_FH_) represent a distinct subset of CD4^+^ T cells specialized in providing help to B lymphocytes, which may play a central role in autoimmune diseases having a major B cell component such as systemic lupus erythematosus. Recently, T_FH_ subsets that share common phenotypic and functional characteristics with T_FH_ cells from germinal centers, have been described in the peripheral blood from healthy individuals. The aim of this study was to analyze the distribution of such populations in lupus patients. Circulating T_FH_ cell subsets were defined by multicolor flow cytometry as T_FH_17 (CXCR3^-^CCR6^+^), T_FH_1 (CXCR3 ^+^ CCR6^-^) or T_FH_2 (CXCR3^-^CCR6^-^) cells among CXCR5 ^+^ CD45RA^-^CD4^+^ T cells in the peripheral blood of 23 SLE patients and 23 sex and age-matched healthy controls. IL-21 receptor expression by B cells was analyzed by flow cytometry and the serum levels of IL-21 and Igs were determined by ELISA tests. We found that the T_FH_2 cell subset frequency is strongly and significantly increased in lupus patients with an active disease (SLEDAI score>8), while the T_FH_1 cell subset percentage is greatly decreased. The T_FH_2 and T_FH_1 cell subset frequency alteration is associated with the presence of high Ig levels and autoantibodies in patient’s sera. Moreover, the T_FH_2 cell subset enhancement correlates with an increased frequency of double negative memory B cells (CD27^-^IgD^-^CD19^+^ cells) expressing the IL-21R. Finally, we found that IgE levels in lupus patients’ sera correlate with disease activity and seem to be associated with high T_FH_2 cell subset frequency. In conclusion, our study describes for the first time the distribution of circulating T_FH_ cell subsets in lupus patients. Interestingly, we found an increased frequency of T_FH_2 cells, which correlates with disease activity. Our results suggest that this subset might play a key role in lupus pathogenesis.

## Introduction

The plasma cell differentiation process essentially takes place in germinal centers (GCs). These structures are mostly made of B cells, which upon antigen-specific interactions with follicular helper T cells (T_FH_ cells) will differentiate into plasma cells or memory B cells. This recently identified subset of CD4^+^ T cells is able to provide help to B cells to undergo proliferation, isotype switching and somatic hypermutation, resulting in long-lasting antibody (Ab) responses [1], mainly through CD40L-CD40 interactions and cytokines [2,3]. T_FH_ cells can migrate to the GC thanks to the CXC chemokine receptor type 5 (CXCR5) and also express Programmed Death-1 (PD-1), Inducible T cell CO-Stimulator (ICOS, especially in humans), the transcription factor B-cell lymphoma 6 (Bcl6) and high levels of interleukin-21 (IL-21). The involvement of T_FH_ cells in shaping the effector function and the fate of B cells, and specially their final differentiation step in plasma cells, implies that they may be central in immune diseases that have a major B cell component. Systemic lupus erythematosus (SLE) is one of these “B-cell mediated” disease, in which hyperactivity of B cells, with excessive production of multiple autoAbs, is perhaps one of the major immunological abnormalities. Indeed, SLE is characterized by the production of antinuclear autoAbs and by the subsequent formation of immune complexes. Some of them play a crucial role in associated cutaneous lesions and glomerulonephritis, which can in turn be fatal [4]. In that context, it was recently shown in our laboratory, that pathogenic autoAbs specific for histone H2B are locally produced by plasma cells, which are detected in the inflamed kidneys of NZB/W lupus mice [5]. Moreover, we demonstrated that the CXCR3 chemokine receptor, that is deeply involved in the inflammatory response and lymphocyte recruitment, is specifically expressed by a subset of freshly differentiated plasma cells, allowing them to migrate to inflamed kidneys where CXCR3 ligands (CXCL9, CXCL10) are produced in excess during renal lupus [6]. Finally, it is clearly admitted that autoAbs and plasma cells are absolutely central to SLE pathogenesis. Indeed, an increased frequency of plasma cell precursors is detected in the blood of children with SLE [7], and the circulating CD27^high^ plasma cell population is expanded in lupus patients and correlates with disease activity [8]. Moreover, a persistent enhancement of plasma cells defined as CD138^+^ cells was recently described even in quiescent SLE patients [9]. Although the role of B cells in disease promotion in lupus has been well established, the precise nature of the CD4^+^ T cells that help autoreactive B cell maturation is less clear. New data suggest that T_FH_ cells are crucial to the pathogenesis of lupus in mice. Dysregulation of T_FH_ cells that promote B cell activation in GCs is associated with the development of SLE in the special roquin san/san mouse model [10,11]. This mouse has a mutation in an enzyme (the ubiquitin ligase roquin) that disrupts a repressor of ICOS, an essential costimulator of T_FH_ cells. Consequences are an excessive number of T_FH_ cells and GC reactions, high levels of IL-21 and the development of a severe SLE-like autoimmune syndrome. The administration of an anti-ICOSL blocking monoclonal Ab (mAb) to lupus NZB/W mice interrupts T_FH_ cell development leading to a decrease in autoAb formation and glomerulonephritis [12,13]. Similar results were obtained in MRL/lpr lupus mice displaying a genetic deletion of ICOS [14]. In addition, abundant T_FH_-like cells are located outside the GC where they support extrafollicular plasmablast maturation and plasma cell differentiation in MRL/lpr and BXSB lupus mice [14,15].

Studies regarding the role of T_FH_ cells in human systemic autoimmune diseases are limited, probably because T_FH_ cells reside into GCs whereas the most available tissue to perform comparative analyses between patients and controls is the peripheral blood. However, a population of CXCR5 ^+^ CD4^+^ T cells within the memory cell compartment (CD45RO^+^) was identified in human peripheral blood [2]. The observation that circulating CXCR5 ^+^ CD4^+^ T cells are diminished in ICOS-deficient humans [16] suggests that they are related to T_FH_ cells. Alterations of circulating CXCR5 ^+^ CD4^+^ T cells have been described in patients with various autoimmune diseases, such as Sjögren’s syndrome, juvenile dermatomyositis, autoimmune thyroid disease and rheumatoid arthritis [17-20]. In lupus, an increase of circulating CD4^+^CXCR5 ^+^ PD-1^high^ T lymphocytes was evidenced in patients with a more severe disease phenotype [21] and a correlation between the expansion of both circulating CXCR5 ^+^ Bcl6 ^+^ CD4^+^ T cells and circulating GC B cells was reported [22]. Taken together, these studies suggest an important role for circulating T_FH_ cells in human autoimmune diseases. However, the function of these cells was not addressed, and their relevance as B-cell helpers in lupus is not fully understood. Moreover, the phenotype of the circulating counterparts of GC T_FH_ remains controversial, as freshly purified CXCR5^+^ and CXCR5^-^ peripheral T cells express comparable levels of ICOS, PD-1 and Bcl6 in the absence of further *ex vivo* activation [18,23,24]. Recently, Morita et al. described a circulating population in the peripheral blood of healthy donors, that shares phenotypic and functional characteristics with T_FH_ cells from GC [18]. Moreover, they distinguished three subclasses, i.e. T_FH_17, T_FH_2 and T_FH_1, defined according to the expression of the CCR6 and CXCR3 chemokine receptors: T_FH_17 cells are CXCR3^-^CCR6^+^ cells whereas T_FH_2 cells are CXCR3^-^CCR6^-^ cells and T_FH_1 cells are CXCR3 ^+^ CCR6^-^ cells. T_FH_17 and T_FH_2 cells were identified as able to provide help to B cells via IL-21 production, resulting in immunoglobulin (Ig) secretion of various isotypes (IgM, IgA, IgG and also IgE for T_FH_2 cells). Moreover, they showed that patients with juvenile dermatomyositis displayed a profound skewing of blood T_FH_ cells towards T_FH_2 and T_FH_17 cells, and this skewing correlated with disease activity, suggesting that an altered balance of T_FH_ subsets contributes to human autoimmunity [18].

In this study, we explored the distribution of T_FH_ cell subsets in relation with disease activity in SLE patients. Our data show that alterations in T_FH_1 and T_FH_2 cell subsets (but not T_FH_17) are strongly associated with an active disease. Moreover, the increased T_FH_2 cell proportion can be related to the presence of high levels of total Ig as well as of anti-double-stranded DNA (ds-DNA) autoAbs in patients’ sera, and to the increase of memory B cells expressing the IL-21 receptor (IL-21R). Finally, the high frequency of T_FH_2 cells seems to be associated with the presence of high IgE levels in sera of active SLE patients.

## Materials and Methods

### Patients and healthy individuals

A total of 111 SLE patients and 63 healthy controls were enrolled in this study. All patients met the American College of Rheumatology criteria for the classification of SLE [25] and disease activity was assessed by the SLE disease activity index (SLEDAI). Routine measures were used to determine anti-nuclear Abs (ANAs, by indirect immunofluorescence with Hep-2 cells) and anti-dsDNA (screened by ELISA; Kallestad anti-DNA microplate EIA, Bio-rad Lab. Inc., CA, USA). All samples were collected from SLE patients undergoing routine evaluation of their disease and from volunteers attending the Strasbourg University Hospitals during routine clinical (diagnosis/prognostic/therapeutic) procedures. Written informed consent was obtained from each individual in agreement with the Helsinky declaration and French legislation (article L1221-8-1), under which no approval by an ethical committee was required in this case.

### Surface staining and flow cytometry analysis

Peripheral blood mononuclear cells (PBMCs) were isolated from heparinized venous blood by centrifugation on Ficoll-Histopaque (Sigma-Aldrich, Saint-Louis, MO, USA). Cells recovered from the gradient interface were washed twice, and stained for 30 min at 4°C with the following Abs or istotype-matched controls: CD3-Alexa700 (UCHT1), CD4-APC (RPA-T4), CXCR5-Alexa488 (RF8B2), CXCR3-PE-Cy5 (1C6), CCR6-PE (11A9), CD19-PerCP-Cy5.5 (HIB19), IgD-FITC (IA6-2), CD27-PE (M-T271), CD38-ECD (HIT2), ICOS-PE (DX29), PD-1-PE-Cy7 (EH12.1), IL-21R-APC (17A12) all from BD Biosciences and CD45RA-ECD (2H4; Beckman Coulter, Fullerton, CA, USA). Cell acquisition was performed using a Gallios cytometer (Beckman Coulter). Data were analyzed with FlowJo (Tree Star) and Kaluza (Beckman Coulter) softwares.

### Intracellular staining and flow cytometry analysis

CD4^+^ T cells were isolated from whole blood using the Human CD4^+^ T cell enrichment cocktail (RosetteSep, StemCell Technologies Inc, Grenoble, France). Briefly, whole blood was incubated with the Ab cocktail 20 min at room temperature, and CD4^+^ T cells were negatively selected following a centrifugation on Ficoll-Histopaque (Sigma-Aldrich). Enriched CD4^+^ T cells (purity>95%) were then stimulated 5h with 25ng/ml phorbol myristate acetate (PMA) and 1µM ionomycin (Sigma-Aldrich) in the presence of monensin (BD GolgiStop™, BD Biosciences, San Diego, CA, USA). Intracellular detection of IL-4 (IL-4-PE; 8D4), IL-17 (IL-17-PE; N49-653), IFN-γ (IFN-γ-APC; B27) (BD Biosciences) and IL-21 (IL-21-APC; 3A3-N2; Miltenyi Biotec, Bergish Gladbach, Germany) was performed on fixed and permeabilized cells according to the manufacturer’s protocol (eBiosiences, San Diego, CA, USA).

### Indirect immunofluorescence

IgE antinuclear antibodies were investigated by indirect immunofluorescence carried out with fixed epithetlial HEp-2 cell slides (Zeus Scientific Inc, NJ, USA). Sera were first depleted of IgG by 1/5 dilution in a purified solution of anti-human IgG sheep IgG (IgG/RF Stripper, AbD Serotec, Kidlington, UK) according to the manufacturer’s instructions. After centrifugation to sediment IgG immune complexes, 25 µl of the IgG-depleted supernatants were incubated with HEp-2 cell smears for 18h at 4°C in a humid chamber. The slides were washed twice with PBS and the cell smears were submitted to a second incubation with 25 µl of mouse IgG anti-human IgE FITC conjugate (Miltenyi Biotech) diluted (1:10) in PBS for 2h at 37°C. After two washes with PBS, slides were mounted with fluorescent mounting medium (DAKO, Gloqtrup, Danemark) and observed with a confocal microscope (LSM 780, Zeiss). The results of the reactions were reported as positive or negative, and the pattern of fluorescence was described as advised for IgG antinuclear antibodies.

### Enzyme-linked immunosorbent assay (ELISA)

The concentration of IgE, IgA and IgG was determined by ELISA in the serum of SLE patients using the human ELISA quantitation set (Bethyl Laboratories, Inc, Montgomery, TX, USA) according to the manufacturer’s instructions. Individual sera diluted at 1:4, 1:50 000 and 1:100 000 for IgE, IgA and IgG respectively were subjected to ELISA analysis, and concentrations in individual samples were calculated according to the standard curve. Each sample was tested in duplicate and results are expressed as mean concentrations ± sem. The detection limit was 30 ng/ml. Serum IL-21 levels were measured by ELISA using the human IL-21 ELISA kit (eBiosciences) according to the manufacturer’s instructions. Results are expressed as the cytokine concentration in pg/ml ± sem and the detection limit was 100 pg/ml. The reactivity of patients’ sera with nucleosome was evaluated by ELISA. Polystyrene plates (Maxisorb, Nunc, Rochester, NY) were coated overnight at 37°C with mouse nucleosomes (1µg/ml as expressed as dsDNA concentration in PBS). Mononucleosome were prepared from L1210 murine cell line as described [26]. They were characterized by 1.5% agarose gel electrophoresis (DNA) and 18% SDS-PAGE (histone content). Pure IgG-depleted sera (see above) were added to plates for 2h, followed by anti-human IgE conjugated to HRP (Bethyl Laboratories). The final reaction was visualized with H2O2 and 3,3’,5,5’-tetramethyl benzidine used as chromogen, and absorbance was measured at 450 nm.

### Statistical analyses

Data were analyzed using Graph Prism version 5 (Graphpad software Inc, San Diego, CA, USA). Differences between SLE patients and healthy individuals were determined with a two-tailed unpaired Student’s test or Mann-Whitney U-test as appropriate. The significance of differences between groups was analyzed with one-way ANOVA test with Bonferroni correction. Relationship between two variables was evaluated using the Spearman’s rank correlation test or Pearson correlation coefficient as indicated. Data are expressed as mean ± sem and differences at p<0.05 or less were considered to be statistically significant.

## Results

### Circulating CXCR5 ^+^ CD4^+^ T cells in SLE patients

As several studies suggested that an increase of circulating T_FH_ cells was associated with autoimmunity, we first analyzed the frequency of CXCR5 ^+^ CD4^+^ T cells and of memory (CD45RA^-^) CXCR5 ^+^ CD4^+^ T cells among total CD4^+^ T cells in 23 consecutive SLE patients (17 with a SLEDAI<5 and 6 with a SLEDAI>8) compared to 23 age- and sex-matched healthy individuals (Table 1). Included patients were untreated or treated with hydroxychloroquine and/or low doses steroids (<20mg/day). All patients who received prolonged and heavy suppressive treatment were excluded from our study.

**Table 1:** Demographic characteristics and circulating CXCR5^+^CD4^+^ T cell frequencies in SLE patients and healthy controls.

	SLE patients (n=23)	Healthy individuals (n=23)	*p* *
Age (years, mean±SD, (range))	40±13 (21-70)	40±12 (22-64)	ns
Female/male	22/1	22/1	
SLEDAI (mean, (range))		na	
inactive disease (n=17; SLEDAI<5)	1.6 (0-3)		
active disease (n=6; SLEDAI>8)	16.8 (12-26)		
CXCR5^+^CD4^+^T cells			
Percentage	13.18±1.2%	12.03±0.9%	ns
Absolute number	70.73±8.31 cells/µl	101.86±9.52 cells/µl	0.02
CXCR5^+^CD45RA^-^CD4^+^T cells			
Percentage	10.16±0.78%	9.18±0.55%	ns
Absolute number	56.57±6.89 cells/µl	79.74±6.52 cells/µl	0.02

na : not applicable, ns: not significant

* *p* values less than 0.05 were considered significant as determined by unpaired t test.

The frequency of CXCR5^+^ cells was not substantially different between SLE patients and healthy controls, neither if analyzed as total CXCR5^+^ T cells (p=0.5), nor if analyzed as memory CD4^+^CD45RA^-^ T cells (p=0.3) (Table 1). On the contrary, the absolute number of CXCR5^+^ T cells is diminished in SLE patients compared to healthy controls (Table 1). This observation most logically reflects the CD4^+^ T cell lymphopenia arising in active SLE patients, which makes difficult and tends to bias the analysis of total T cell populations. We also confirmed that typical markers for GC T_FH_ cells such as PD-1 and ICOS do not discriminate between CXCR5^-^ and CXCR5^+^ cells in the absence of *ex vivo* activation between CXCR5^-^ and CXCR5^+^ cells (Figure S1). Therefore, we decided to focus our analysis on the polarization of circulating T_FH_ cells toward Th1, Th2 and Th17 phenotypes and studied the distribution of these T_FH_ subsets within the CXCR5 ^+^ CD4^+^ T cell pool in SLE patients.

### Definition of T_FH_ cell subsets

**Figure 1:**
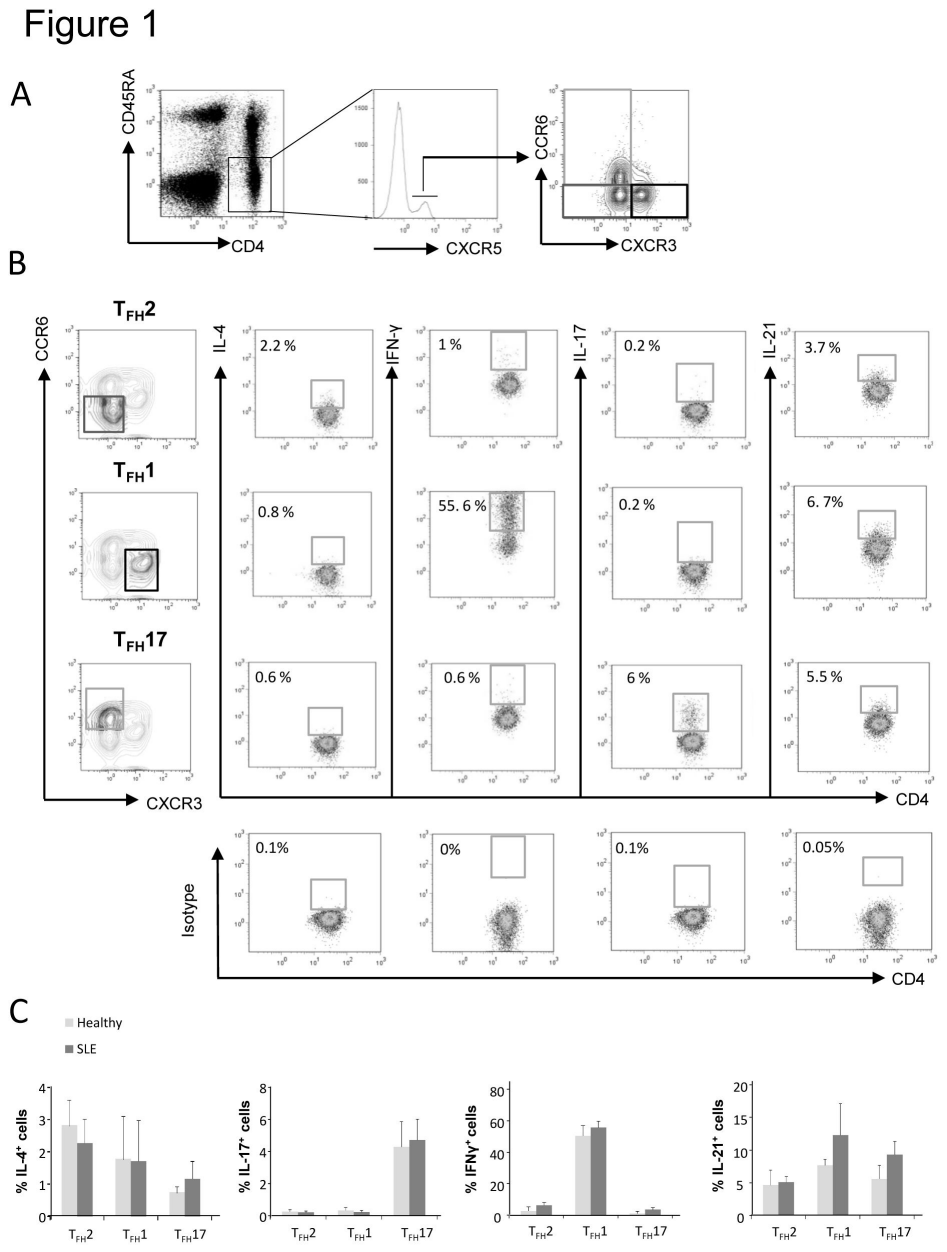
Cytokine production by T_FH_ cell subsets. Purified CD4^+^ T cells from healthy controls were cultured for 5h with PMA, ionomycin and monensin. CD4^+^ T cells were then stained with fluorescently-labeled Abs specific for CD4, CD45RA, CXCR5, CCR6 and CXCR3. (A) T_FH_ subset distribution was determined thanks to CXCR3 and CCR6 expression on gated CD4^+^CD45RA^-^CXCR5^+^ T cells allowing the identification of T _FH_17 cells (CXCR3^-^CCR6^+^, light grey), T _FH_2 cells (CXCR3^-^CCR6^-^, dark grey) and T _FH_1 cells (CXCR3 ^+^ CCR6^-^, thick black). Frequencies of IL-4, IFN-γ, IL-17 and IL-21 positive cells were determined by intracellular staining on each T_FH_ subset after setting the threshold using isotype control staining. Dot plots (B) from one healthy control is shown as example and histograms (C) from 3 healthy controls and 3 SLE patients are shown. Data are expressed as % ± sem.

As described by Morita and colleagues, differential expression of CXCR3 and CCR6 chemokine receptors defines three major subsets within blood CXCR5 ^+^ CD4^+^ T cells [18]. Based on this unique but extremely interesting study, we thus analyzed the expression of CXCR3 and CCR6 on CD4^+^CD45RA^-^CXCR5^+^ circulating T cells and the gating strategy used to identify each T_FH_ subset by flow cytometry is represented in Figure 1A. In order to more precisely define these T_FH_ subsets, we assessed the intracellular expression of cytokines typically associated with Th2, Th1 and Th17 phenotype (i.e. IL-4, IFN-γ and IL-17) as well as IL-21, following PMA/ionomycin stimulation. As expected, we found that the T_FH_2, T_FH_1 and T_FH_17 subsets from healthy individuals and SLE patients, produce IL-4, IFN-γ and IL-17 respectively (Figure 1B-C). Regarding IL-21, we found that it was produced by T_FH_2, T_FH_1 and T_FH_17 subsets (Figure 1B-C). Taken as a whole, our *ex vivo* definition of T_FH_ cell subsets is consistent with the results obtained by Morita and colleagues upon long-term *in vitro* stimulation of circulating CXCR5^+^ T_FH_ cells [18].

### T_FH_ cell subset frequencies in SLE patients

**Figure 2:**
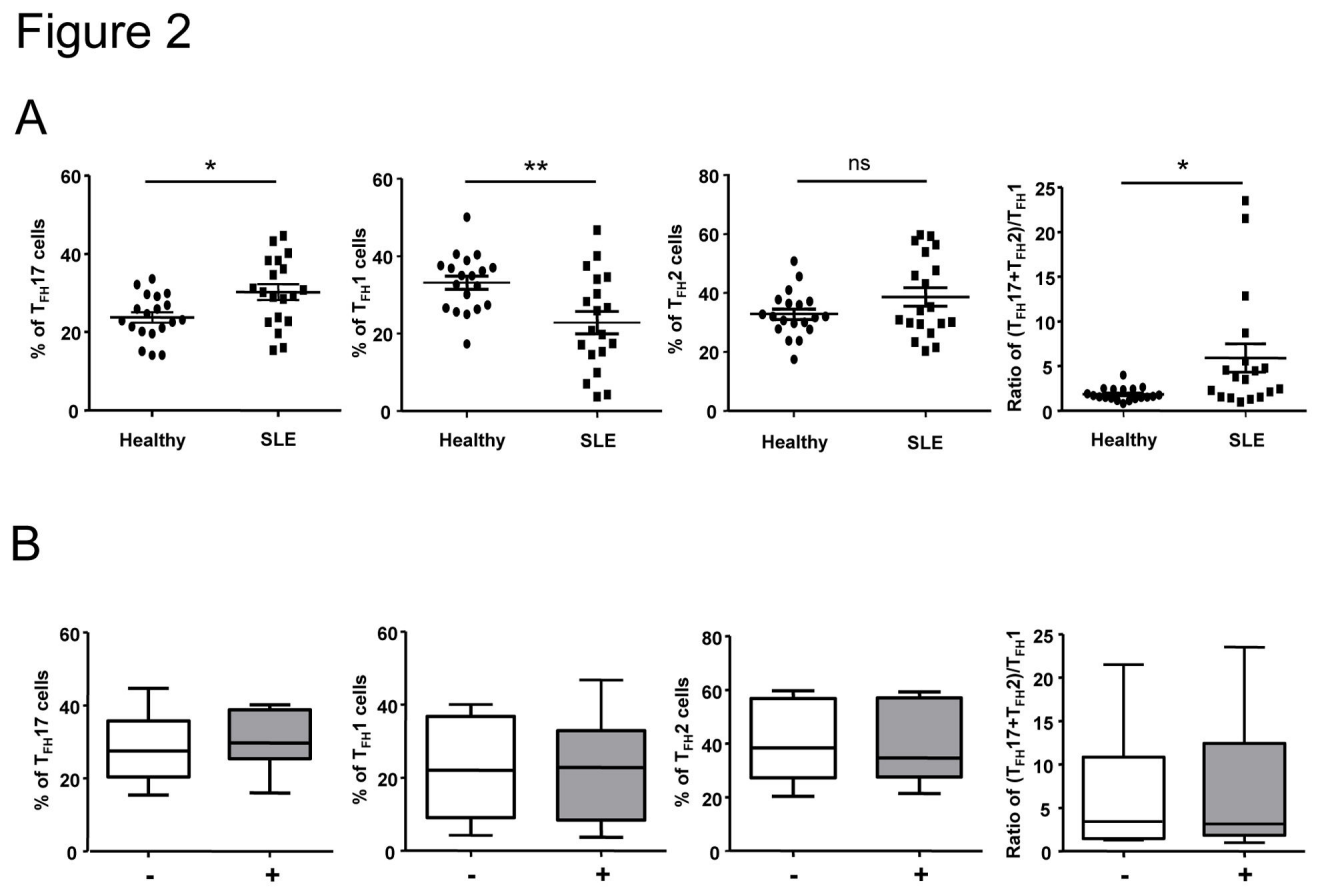
T_FH_ cell subset distribution is altered in SLE patients. PBMCs from SLE patients (n = 19) and sex and age-matched healthy controls (n = 19) were stained for CD4, CD45RA, CXCR5, CXCR3 and CCR6. The frequency of each T_FH_ cell subset as well as the calculated (T _FH_17+T _FH_2)/T _FH_1 ratio are represented (A). Each data point represents an individual subject; horizontal lines show the mean ± sem. Percentages of each T_FH_ cell subset and the ratio of (T _FH_17+T_FH_2)/T_FH_1 cells in SLE patients according to steroid treatment (-, white box, n = 8 and +, grey box, n = 6) are represented (B). *p < 0.05, **p < 0.01 (Mann-Whitney U test).

We thus wondered whether the frequency of T_FH_ subsets was altered in SLE patients, and we analyzed the blood CXCR5^+^ T_FH_ subset distribution in our cohort of 23 SLE patients and age- and sex-matched healthy individuals (Table 1). As shown in Figure 2A, the frequency of T_FH_17 cells within CD4^+^CD45RA^-^CXCR5^+^ T cells was significantly higher in SLE patients compared to healthy individuals (30±2% *vs* 23.8±2%; p<0.05, n=19). In contrast, the frequency of T_FH_1 cells within CD4^+^CD45RA^-^CXCR5^+^ T cells was significantly lower in SLE patients compared to healthy individuals (22.8±2.9% *vs* 33.2±1.7%; p<0.01, n=19). No significant difference of T_FH_2 frequency was observed, however the ratio of T_FH_2+T_FH_17 (defining B helper T cells) over T_FH_1 (non B helper T cells) was significantly enhanced in SLE patients compared to healthy individuals (5.9±1.6% *vs* 1.9±0.2%; p<0.05, n=19; Figure 2A). Neither T_FH_17 frequency enhancement, nor T_FH_1 frequency diminution were due to treatments as the frequencies observed in patients receiving or not hydroxychloroquine (data not shown) or prednisolone (Figure 2B) were equivalent.

### T_FH_ subset frequencies in active versus inactive SLE patients

**Figure 3:**
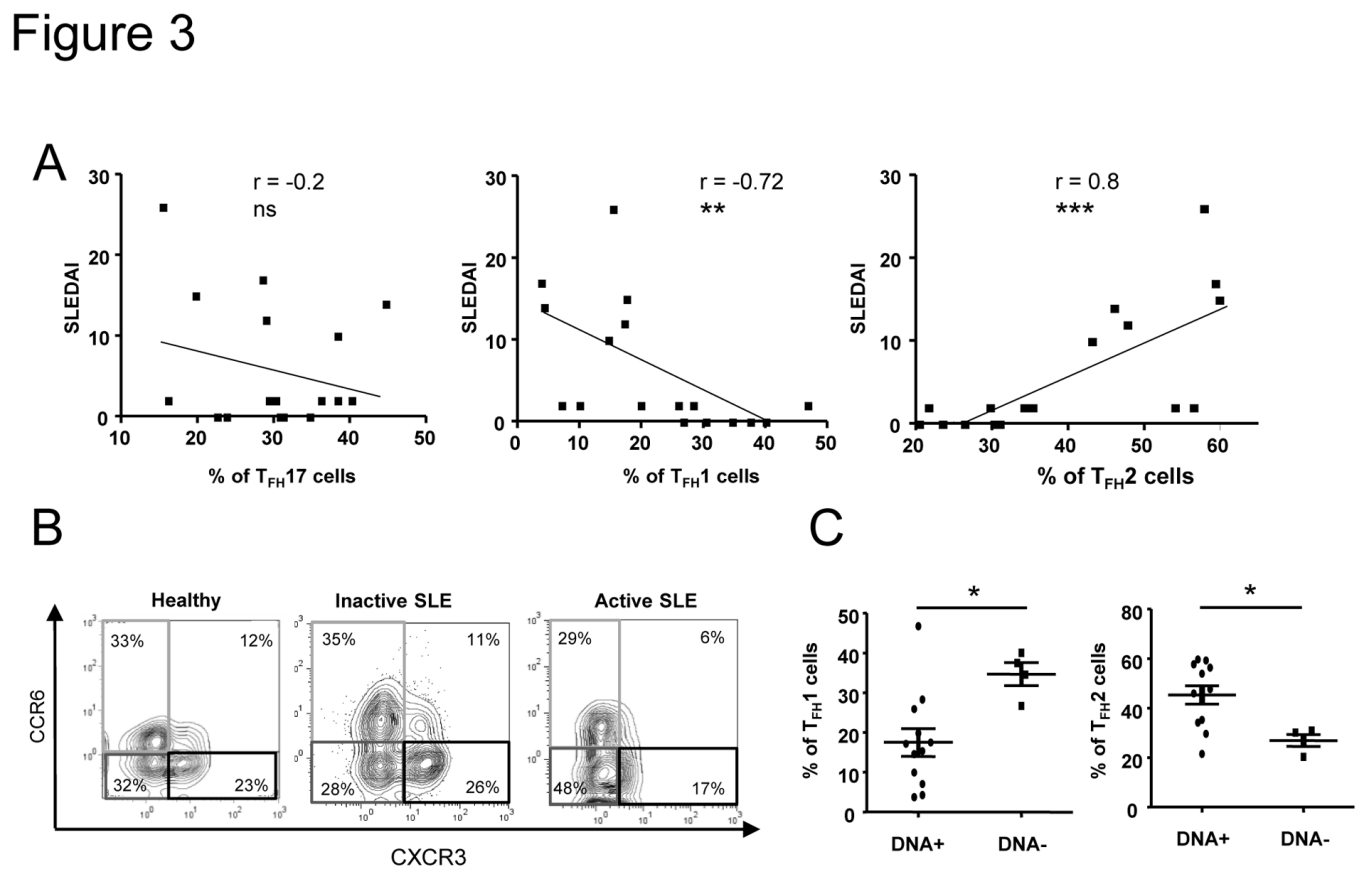
T_FH_2 cell frequency is significantly increased in active SLE patients. Correlations between the SLEDAI score and the percentage of each T_FH_ cell subset from SLE patients (n = 19) are shown (A). Dot plots of CXCR3 and CCR6 expression on gated CD3^+^CD4^+^CD45RA^-^CXCR5^+^ T cells from an healthy individual, an inactive SLE patient and an active patient are shown as examples (B). T_FH_2 and T_FH_1 cell subset frequencies were analyzed according to the presence or the absence of anti-dsDNA Abs (C). Each data point represents an individual subject; horizontal lines show the mean ± sem. *p < 0.05, **p < 0.01, ***p < 0.001 (unpaired Student’s t test and Spearman’s rank correlation test). ns: not significant.

To determine whether the altered T_FH_ subset frequencies were associated with disease activity, we then analyzed the frequency of each T_FH_ subset in relation with the SLEDAI score. Interestingly, and contrary to what we expected, the T_FH_17 cell frequency was not associated with disease activity (p=0.4; Figure 3A). However, the T_FH_2 cell frequency was strongly and significantly correlated (r=0.79; p=0.0002), while the T_FH_1 cell frequency was inversely associated with the SLEDAI score (r=-0.73; p=0.001; Figure 3A). Indeed, when patients were sub-grouped according to disease activity (Figure S2), T_FH_17 cell frequency was significantly but only fairly increased in patients with an inactive disease compared to healthy individuals but not to SLE patients with an active. However, T_FH_2 cell frequency was highly enhanced in patients with an active disease (52.2±3.1%) compared to healthy individuals (32.8±1.8%, p<0.001) or to SLE patients with an inactive disease (32.4±3.1%, p<0.001; Figure 3B is shown as a representative example). Concerning T_FH_1 cell frequency, patients with a high SLEDAI score displayed 12.1±2.1% of T_FH_1 cells, while T_FH_1 cells in healthy individuals or SLE patients with an inactive disease represented 33.2±1.7% (p<0.0001) and 27.8±3.2% (p<0.01) of CXCR5 ^+^ CD45RA^-^CD4^+^ T cells respectively (Figure S2).

As T_FH_ cells play a critical role in the development of Ag-specific humoral responses, we investigated the relationship between anti-dsDNA autoAbs, which are characteristic for lupus, and T_FH_ cell subset distribution in SLE. We found that T_FH_1 and T_FH_2 cell frequency deviations were significantly related to the presence of anti-dsDNA autoAbs in the serum of SLE patients (17.8±3.8% of T_FH_1 cells and 45.5±4% of T_FH_2 cells in the group of patients harboring anti-dsDNA autoAbs *vs* 33.8±2.4% of T_FH_1 cells and 26.2±2% of T_FH_2 cells in the group of patients without anti-dsDNA autoAbs, p=0.015; Figure 3C). Taken together, our results indicate that alterations in T_FH_1 and T_FH_2 cell frequencies are associated with disease activity in lupus.

### CD4^+^CXCR5^-^ T_H_ cell frequencies in SLE patients

**Figure 4:**
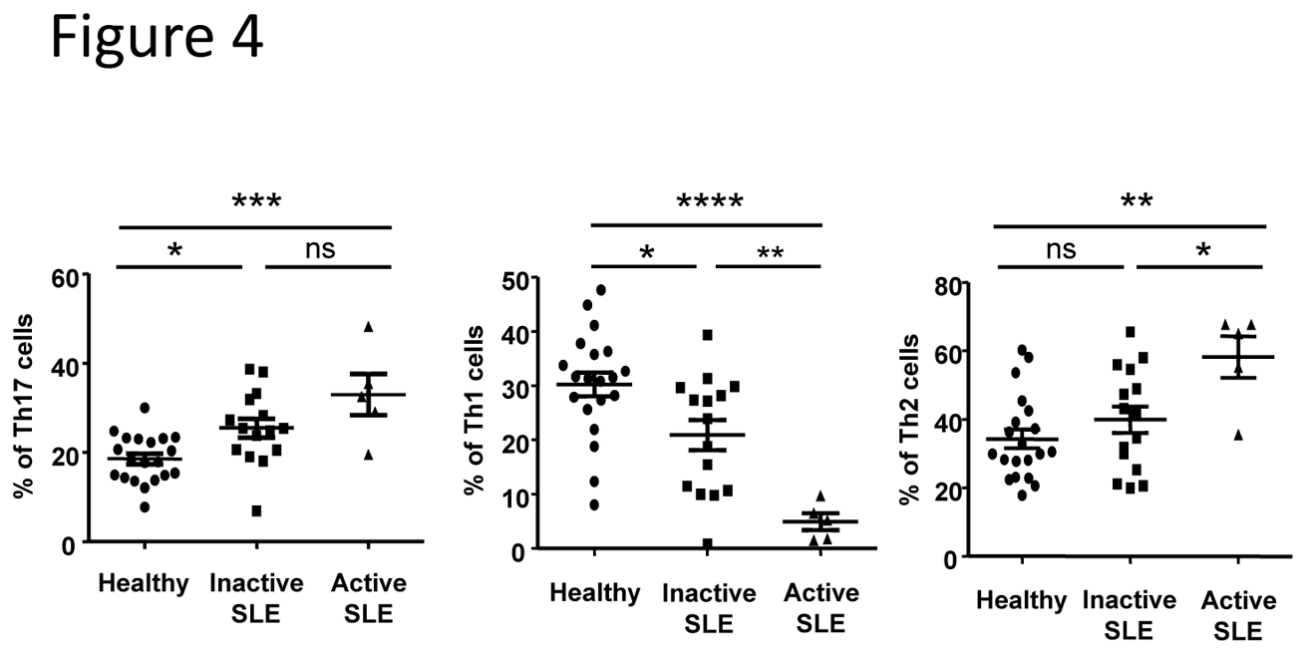
Th cell subset distribution is altered in SLE patients. Th cell subset frequencies were determined according to the expression of CXCR3 and CCR6 on CD4^+^CD45RA^-^CXCR5^-^ T cells. The frequency of each Th cell subset was defined in samples from inactive SLE patients (n = 13), active SLE patients (n = 6) and sex and age-matched healthy controls (n = 19). Results are expressed as the mean ± sem. *p < 0.05, **p < 0.01, ***p < 0.001 (one-way ANOVA test). ns: not significant.

As we did not observe any T_FH_17 cell frequency alteration whereas recent data suggest an important role of IL-17 secreting cells in SLE pathogenesis, we analyzed the frequency of Th1, Th2 and Th17 cells within the CD45RA^-^CXCR5^-^CD4^+^ T cell compartment. Both alterations observed for CXCR3 ^+^ CCR6^-^ T cells (Th1 phenotype) and CXCR3^-^CCR6^-^ T cells (Th2 phenotype) within CXCR5 ^+^ CD4^+^ T cells also occurred within CXCR5^-^CD4^+^ T cells (Figure 4). On the other hand, whereas no significant expansion of CXCR3^-^CCR6^+^ T cells (Th17 phenotype) was found within CXCR5 ^+^ CD4^+^T cells in patients with an active disease (Figure 3B), Th17 cell frequency was highly significantly increased in active SLE patients (33±4.7% *vs* 18.6±1.2% in healthy individuals, p< 0.001; Figure 4).

### High T_FH_2 cell subset frequency correlates with increased IL-21R expressing memory B cells

**Figure 5:**
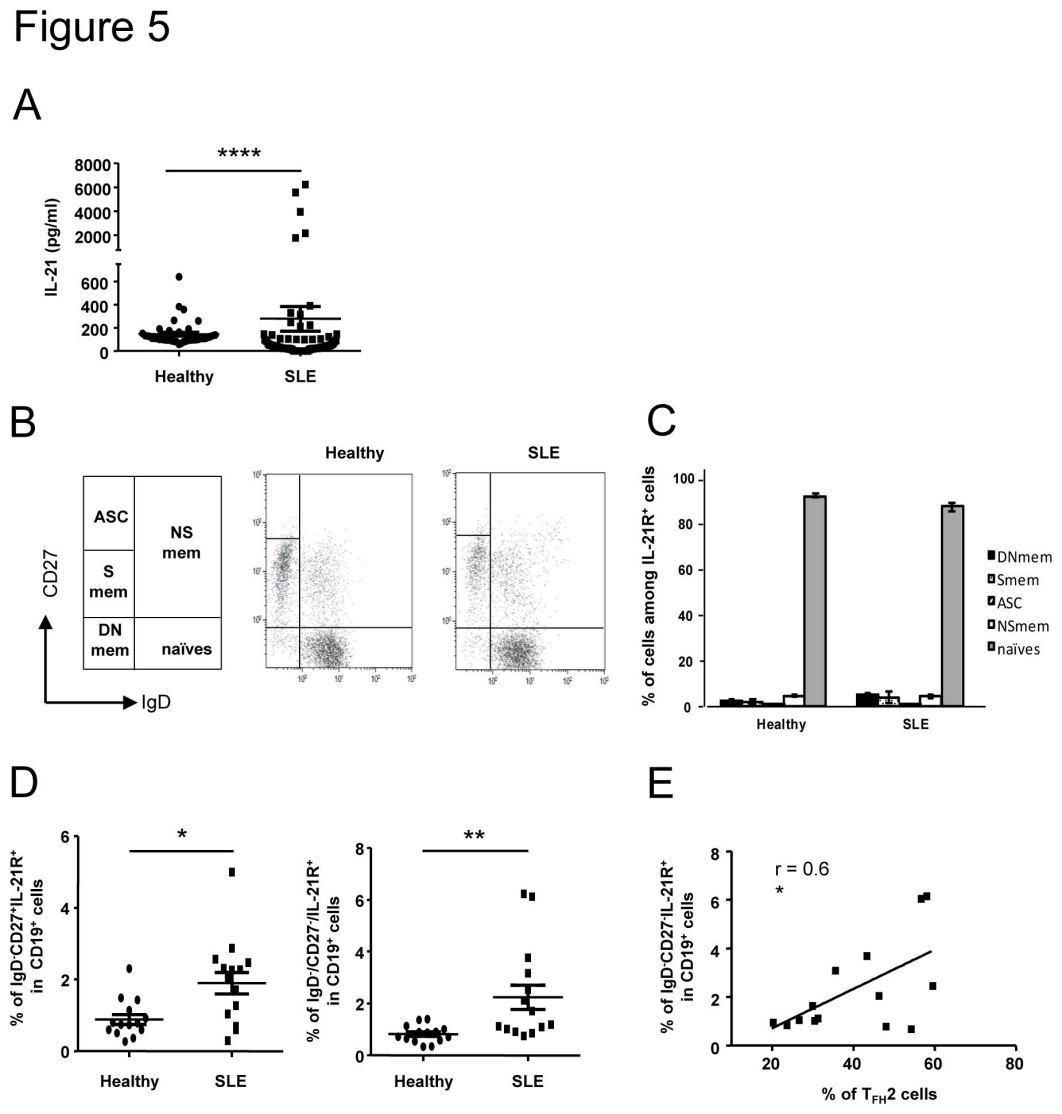
The frequency of memory B cells expressing IL-21R is enhanced in SLE patients and correlates with T_FH_2 cell increase. IL-21 concentration was measured in the serum of SLE patients (n = 88) and healthy controls (n = 44) by a sandwich ELISA assay (A). Results are expressed as the mean IL-21 concentration (pg/ml) from duplicate wells. Each data point represents an individual subject and horizontal lines show the mean ± sem. Circulating B cell subsets were defined according to the expression of IgD and CD27 on CD19^+^IL-21R^+^ cells allowing the definition of 5 populations: Ab-secreting cells (ASC), non switched memory cells (NSmem), switched memory cells (Smem) and double negative memory cells (DNmem). Representative dot plots obtained with samples from one healthy control and one SLE patient are shown as an example (B). The distribution of each B cell subset among IL-21R^+^ cells in healthy controls (n = 14) and SLE patients (n = 14) is represented (C). The frequency of CD27^+^ memory (left) and DN memory (right) B cells expressing IL-21R is compared between healthy controls (n = 14) and SLE patients (n = 14). Correlation between the IL-21R^+^ DN memory cells and T_FH_2 cell subset frequencies in SLE patients (n= 14) is represented (E). Each data point represents an individual subject; horizontal lines show the mean ± sem. *p < 0.05, **p < 0.01 (Mann-Whitney U test and Pearson correlation coefficient).

T cell help to B cells proceeds through cell-cell interactions and secreted cytokines. A major cytokine produced by T_FH_ cells and involved in B cell help is IL-21 [27]. That’s why we decided to determine the concentration of IL-21 in the serum of a large cohort of SLE patients. As described by other groups [28,29], we observed that the concentration of IL-21 was significantly higher in sera from SLE patients than in those of healthy individuals (278±106 pg/ml in SLE patients, n=88 *vs* 146±17 pg/ml in healthy individuals, n=40, p<0.0001; Figure 5A). To better define T_FH_-B cell dialogue in lupus, we investigated the expression of IL-21R by B cells analyzed by three-color cytometry using anti-CD19, anti-CD27 and anti-IgD Abs. This allows the identification of five well-defined B cell subsets (Figure 5B) i.e. mature naïve B cells (IgD ^+^ CD27^-^), Ab-secreting cells (ASC; IgD^-^CD27^hi^), switched memory B cells (Smem; IgD^-^CD27^+^), double negative memory B cells (DN; IgD^-^CD27^-^, a population of memory B cells described to be enhanced in lupus patients [30]) and non-switched memory B cells (NSmem; IgD ^+^ CD27^+^). As previously described, the IL-21R is mostly expressed by mature naïve CD19^+^ B cells (around 90% of gated IL-21R^+^ cells are CD19^+^IgD ^+^ CD27^-^), not only in healthy individuals but also in SLE patients (92.1±3.4% in healthy individuals *vs* 87.5±7.1% in SLE patients; Figure 5C). However, we found a significant increase of memory B cells expressing IL-21R cells among CD19^+^ B cells in lupus patients compared to healthy individuals. This increase concerns both CD27^+^ memory B cells (1.9±0.3% *vs* 0.9±0.1% of IgD^-^CD27^+^IL-21R^+^ B cells, p=0.01, n=15) and DN memory B cells (2.2±0.5% *vs* 0.8±0.1% of IgD^-^CD27^-^IL-21R^+^ B cells, p=0.002, n=15; Figure 5D). Interestingly, the frequency of DN memory B cells expressing IL-21R was correlated to T_FH_2 cell frequency in SLE patients (r=0.6, p=0.026; Figure 5E).

### High levels of IgE in the serum of SLE patients with an active disease

**Figure 6:**
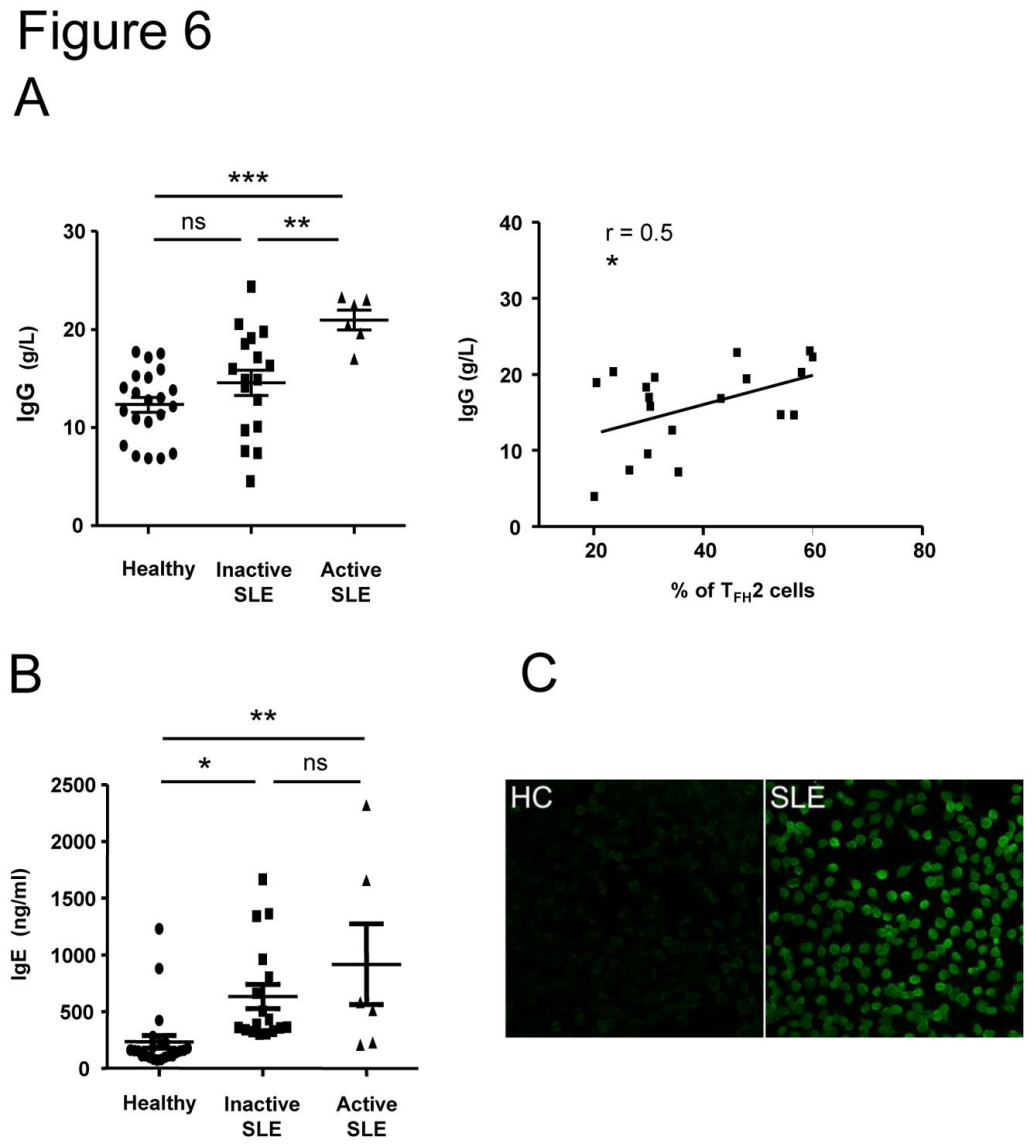
IgE levels are increased in the serum of SLE patients. The quantification of IgG and IgE levels in the serum of inactive SLE patients (n = 17), active SLE patients (n = 6) and sex and age-matched healthy controls (n = 21) were determined by a sandwich ELISA assay. Results are expressed as the mean IgG concentration (g/L; A) or IgE concentration (ng/ml; B) from duplicate wells and correlation between IgG levels and T_FH_2 cell frequency is represented (A). Each data point represents an individual subject and horizontal lines show the mean ± sem. * *p* < 0.05, **p < 0.01 (unpaired Student’s t test and Pearson correlation coefficient). ns: not significant. ANA IgE in sera were detected using Hep-2 cells as substrate (C). Compared to healthy individual sera (HC, shown as an example), some IgG-depleted sera from lupus patients (4/12) (SLE, 1 out of 4 representative picture) yielded homogeneous nuclear staining.

Both T_FH_2 and T_FH_17 subsets were described as able to help B cells to differentiate into plasma cells and to produce Igs [18]. Accordingly to enhanced T_FH_2 (and T_FH_17) cell frequency, SLE patients displayed higher serum IgG (Figure 6A) and IgA (not shown) levels than did healthy individuals. Moreover, the increased IgG concentration in lupus sera correlated with higher T_FH_2 cell frequency (Figure 6A, right). The main difference between these two subsets concerns their capacity to induce IgE production, as the T_FH_2 cell subset is the only one able to help B cells to secrete IgE. We then measured the serum IgE levels and we observed a significant increase of IgE concentrations in SLE patients (n=23, 17 patients with a SLEDAI score<5 and 6 patients with a SLEDAI score>8) compared to sera from healthy individuals (n=23; 233±57 pg/ml), and this increase was more pronounced in patients with an active disease (917±355 pg/ml in patients with active SLE, p<0.01 and 633±106 pg/ml in patients with inactive SLE, p<0.05; Figure 6B). We next wondered whether IgE Abs from lupus patients could be specific for nuclear Ag and we thus investigated the occurrence of antinuclear IgE autoAbs in IgE^+^ sera from SLE patients (n=12) and healthy controls (n=3). Antinuclear IgE Abs were detected by indirect immunofluorescence in SLE patients only (4/12, 33%; Figure 6C), and among these 4 SLE patients’ sera, 2 were also found to be reactive with nucleosome as determined by ELISA (data not shown).

## Discussion

T_FH_ cells are crucial immune regulators and have been implicated in the pathogenic processes, which occur in many human diseases. The present study investigates the frequency of peripheral T_FH_ cells in SLE and describes for the first time the distribution of T_FH_1, T_FH_2 and T_FH_17 cell subsets according to disease activity. Our results demonstrate that the frequency of the T_FH_2 cell subset is significantly increased in SLE patients with an active disease compared to patients with an inactive disease or to healthy controls, while the frequency of the T_FH_1 cell subset (unable to provide B cell help) is significantly decreased in active SLE patients. The functional significance of this T_FH_2/T _FH_1 unbalance still remains unclear and additional studies are required to define it more precisely. Migration of the T_FH_1 cell subset into inflamed organs thanks to CXCR3 expression may participate to the T_FH_1 cell subset frequency diminution in the peripheral blood, and thus to the T_FH_2 cell frequency enhancement. Indeed, CXCR3 bearing CD4^+^T cells infiltrating the inflamed kidneys have been detected in patients with active lupus nephritis [31]. However, we should keep in mind that in SLE patients, we did not find any significant diminution of CXCR5^+^ circulating T cells expressing both CCR6 and CXCR3. Immunostainings on renal biopsies from SLE patients will certainly help to answer this question. Moreover, we demonstrated that the high T_FH_2 and low T_FH_1 frequencies are associated with typical lupus-related biological parameters such as high Ig and anti-dsDNA levels, and the presence of IgE, whereas the proportion of the T_FH_17 cell subset is not associated with disease activity. This result is surprising as several studies suggested the involvement of Th17 cells in lupus pathogenesis [32,33] and an increased T_FH_17 cell frequency has been shown to be correlated with disease activity in other autoimmune syndromes [17,18]. However, we found that the frequency of regular (CXCR5^-^) Th17 cells rather than of (CXCR5^+^) T_FH_ 17 cells was significantly increased in SLE patients with an active disease. Taken together, our data provide the first evidence that T_FH_ subsets display an altered balance in SLE, which may be involved in the pathogenesis of this disease.

With the aim of defining T_FH_ and B cell interactions, we analyzed the expression of IL-21R on lupus B cells. We found an increase of memory B cells expressing IL-21R in SLE patients. Interestingly, this result was only observed within “switched” memory B cells (both CD27^+^ and CD27^-^ cells) but not within the “non-switched” CD27^+^IgD ^+^ IgM^+^ population, which is known to be diminished in SLE [9,34]. Interestingly, we observed a positive correlation between the frequency of T_FH_2 cells and DN memory B cells expressing IL-21R (and not CD27^+^IL-21R^+^ memory B cells). A detailed analysis of this DN memory cell population revealed that correlation between DN memory B cells and disease activity in lupus is restricted to DN memory B cells with an activated phenotype (CD95^+^) [35]. This activation state could thus explain the higher IL-21R expression on DN memory B cells, as memory B cells up-regulate the IL-21R following activation [36].

The fine relationship between T_FH_2 cells and DN memory B cells in active lupus is not understood, but one can speculate that the expansion of these two major partners involved in the final Ig production is critical in lupus pathogenesis. Indeed, the T_FH_2 cell subset was clearly demonstrated to be able to promote B cell differentiation into Ab-secreting cells, notably through IL-21 secretion [18]. Moreover, the ability of DN memory B cells to differentiate into plasma cells is not known, but their higher IL-21R expression as well as previous description of human post-switched IgG^+^ memory B cells specifically and exclusively sensitive to IL-21 and BAFF and capable of rapidly differentiating into plasma cells [37], lead us to postulate that the DN memory population could also highly respond to IL-21 (potentially associated to other unknown factors) thereby resulting in the generation of more plasma cells and in much greater amounts of secreted Igs. Interestingly, Morita and colleagues demonstrated that T_FH_2 cells are more efficient in helping memory B cells to produce Igs than T_FH_17 cells [18]. Further studies are required to validate this hypothesis and we are currently investigating the ability of T_FH_2 cells to induce DN memory B cell differentiation into plasma cells through IL-21.

In the present work, we found that the frequency of circulating T_FH_2 cells is associated with the presence of anti-dsDNA autoAbs, which role in SLE is clearly validated both in animals and humans. A large amount of these anti-dsDNA autoAbs consist in high affinity IgG, and interestingly, we also observed that increased T_FH_2 cell frequency is related to a high concentration of IgG in patients’ sera. More importantly, recent data suggest a role for IgE autoAbs in lupus pathogenesis, as antinuclear IgE Abs (reacting with nucleosomes and dsDNA) are detected in the serum of SLE patients, without associated allergy [38]. Moreover, anti-dsDNA IgE levels are highly associated with active lupus nephritis and total IgE levels correlate with disease activity [39]. We also measured high concentrations of IgE in sera of SLE patients, particularly in sera from patients with an active disease who also exhibit a greatly enhanced T_FH_2 cell frequency. Of note, functional analysis of T_FH_ subsets by Morita et al. demonstrated that T_FH_2 cells are the only ones able to induce differentiation of IgE-producing plasmablasts [18]. Interestingly, we observed the existence of autoreactive IgE (anti-nuclear Abs- and anti-nucleosome Abs) in patients’ sera and particularly in those from patients with active lupus and high T_FH_2 levels. These observations suggest that autoreactive IgE may play a role in SLE pathogenesis, however, factors influencing IgE production are multiple and further studies are required to better define IgE implication in lupus.

We are aware of the fact that our study certainly has some limitations. Our overall sample size is small and especially the number of patients with an active disease is limited, notably because we decided to include in this study only patients with no or low medications. Indeed, it was recently demonstrated that high-doses of corticosteroid treatments down-regulate circulating CXCR5 ^+^ PD-1 ^+^ CD4^+^ T cell frequencies [40]. However, it is remarkable that in this group of six patients with an active disease, the T_FH_2 frequency was strongly and systematically enhanced. Studies in larger cohorts will help to validate this observation and co-culture experiments of purified T_FH_ subsets and B cells will provide additional functional evidences.

## Conclusions

In conclusion, we found an increased proportion of the T_FH_2 cell subset in SLE patients with an active disease. This increase is associated with key biological SLE parameters (total Ig levels and anti-dsDNA Abs), with B cell subset alterations and with the presence of high IgE levels. Our results provide new insights and a rationale for studying circulating T_FH_ subsets in systemic autoimmune diseases and are crucial in view of defining new targets for therapeutic interventions.

## Supporting Information

Figure S1 Peripheral CXCR5^-^ and CXCR5^+^ CD4^+^ T cells express similar levels of PD-1 and ICOS. Surface expression of PD-1 and ICOS molecules was analyzed on circulating CXCR5^-^ and CXCR5^+^ CD4^+^CD45RA^-^ T cells by FACS. Staining with the corresponding isotype control Ab is shown (grey shaded areas) and the mean fluorescence intensity is indicated in each histogram. Representative data from 3 independent experiments are shown.
(PPT)

Figure S2 T_FH_ cell subset distribution in active SLE patients. T_FH_ cell subset distribution in active SLE patients (n = 6), inactive SLE patients (n = 13), and sex and age-matched healthy controls (n = 19) is represented. Each data point represents an individual subject; horizontal lines show the mean ± sem. *p < 0.05, **p < 0.01, ***p < 0.001, ****p < 0.0001 (one-way ANOVA test). ns: not significant.
(PPT)
