# Supplementary figures and images for: Circulating TFH Subset Distribution Is Strongly Affected in Lupus Patients with an Active Disease
Source: PLoS One. 2013 Sep 19;8(9):e75319. doi: 10.1371/journal.pone.0075319 (PMC3777901; doi:10.1371/journal.pone.0075319)

## Slide 1
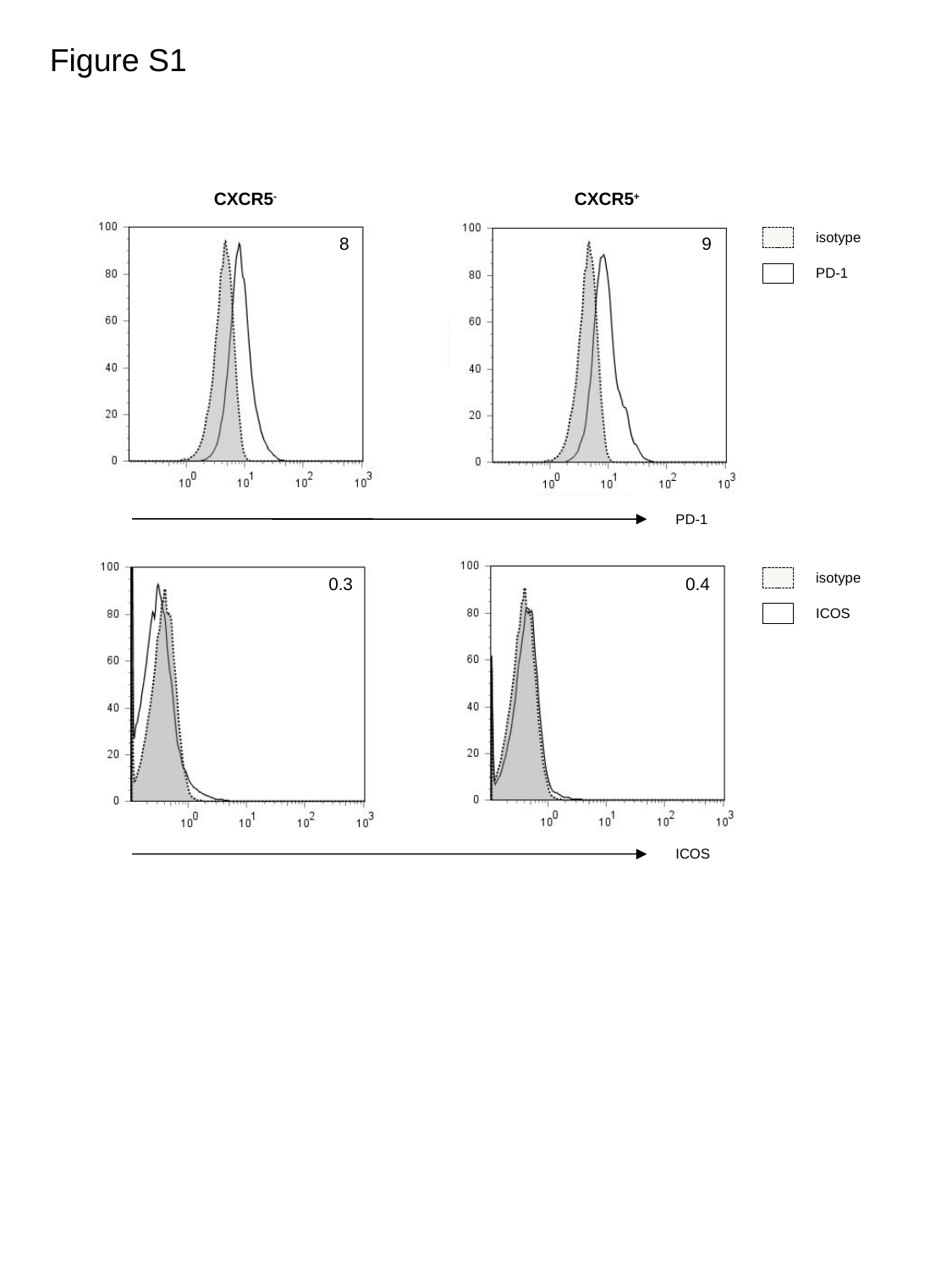

Figure S1
CXCR5-
CXCR5+
isotype
8
9
PD-1
PD-1
isotype
0.3
0.4
ICOS
ICOS

Supplement: Figure S1 — Peripheral CXCR5- and CXCR5+ CD4+ T cells express similar levels of PD-1 and ICOS. Surface expression of PD-1 and ICOS molecules was analyzed on circulating CXCR5- and CXCR5+ CD4+CD45RA- T cells by FACS. Staining with the corresponding isotype control Ab is shown (grey shaded areas) and the mean fluorescence intensity is indicated in each histogram. Representative data from 3 independent experiments are shown. (PPT) [file pone.0075319.s001.ppt]
