# Supplementary material for: Circulating TFH Subset Distribution Is Strongly Affected in Lupus Patients with an Active Disease
Source: PLoS One. 2013 Sep 19;8(9):e75319. doi: 10.1371/journal.pone.0075319 (PMC3777901; doi:10.1371/journal.pone.0075319)
Supplement: Figure S2 — TFH cell subset distribution in active SLE patients. TFH cell subset distribution in active SLE patients (n = 6), inactive SLE patients (n = 13), and sex and age-matched healthy controls (n = 19) is represented. Each data point represents an individual subject; horizontal lines show the mean ± sem. *p < 0.05, **p < 0.01, ***p < 0.001, ****p < 0.0001 (one-way ANOVA test). ns: not significant. (PPT) [file pone.0075319.s002.ppt]

## Slide 1
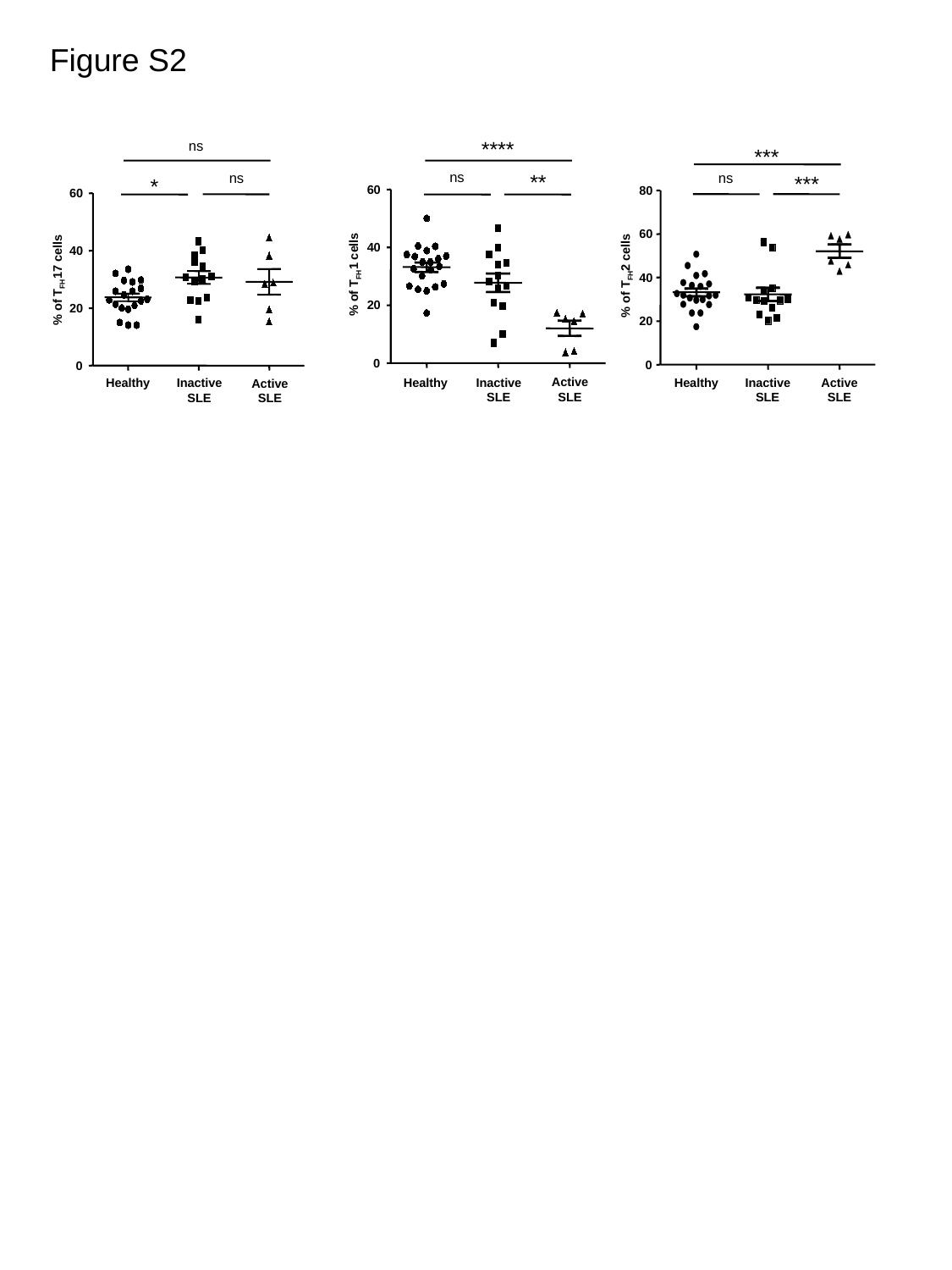

Figure S2
****
ns
***
ns
***
80
60
% of TFH2 cells
40
20
0
Healthy
Inactive
SLE
Active
SLE
ns
**
ns
60
40
% of TFH1 cells
20
0
Active
SLE
Healthy
Inactive
SLE
*
60
40
% of TFH17 cells
20
0
Healthy
Inactive
SLE
Active
SLE
